# Convergent Amino Acid Signatures in Polyphyletic *Campylobacter jejuni* Subpopulations Suggest Human Niche Tropism

**DOI:** 10.1093/gbe/evy026

**Published:** 2018-02-14

**Authors:** Guillaume Méric, Alan McNally, Alberto Pessia, Evangelos Mourkas, Ben Pascoe, Leonardos Mageiros, Minna Vehkala, Jukka Corander, Samuel K Sheppard

**Affiliations:** 1 Department of Biology and Biochemistry, The Milner Centre for Evolution, University of Bath, United Kingdom; 2 Institute of Microbiology and Infection, University of Birmingham, United Kingdom; 3 Department of Mathematics and Statistics, University of Helsinki, Finland; 4 Department of Biostatistics, University of Oslo, Norway; 5 Pathogen Genomics, Wellcome Trust Sanger Institute, Hinxton, Cambridgeshire, United Kingdom

**Keywords:** *Campylobacter*, phylogenetics, adaptation, pathogenesis, human niche

## Abstract

Human infection with the gastrointestinal pathogen *Campylobacter jejuni* is dependent upon the opportunity for zoonotic transmission and the ability of strains to colonize the human host. Certain lineages of this diverse organism are more common in human infection but the factors underlying this overrepresentation are not fully understood. We analyzed 601 isolate genomes from agricultural animals and human clinical cases, including isolates from the multihost (ecological generalist) ST-21 and ST-45 clonal complexes (CCs). Combined nucleotide and amino acid sequence analysis identified 12 human-only amino acid KPAX clusters among polyphyletic lineages within the common disease causing CC21 group isolates, with no such clusters among CC45 isolates. Isolate sequence types within human-only CC21 group KPAX clusters have been sampled from other hosts, including poultry, so rather than representing unsampled reservoir hosts, the increase in relative frequency in human infection potentially reflects a genetic bottleneck at the point of human infection. Consistent with this, sequence enrichment analysis identified nucleotide variation in genes with putative functions related to human colonization and pathogenesis, in human-only clusters. Furthermore, the tight clustering and polyphyly of human-only lineage clusters within a single CC suggest the repeated evolution of human association through acquisition of genetic elements within this complex. Taken together, combined nucleotide and amino acid analysis of large isolate collections may provide clues about human niche tropism and the nature of the forces that promote the emergence of clinically important *C. jejuni* lineages.

## Introduction

Many bacterial species that are known as causes of gastroenteritis are common commensal organisms causing little or no harm to the host species. For pathogenic strains of these species, the pathway to disease can involve a series of population bottlenecks. Therefore, clinical isolates sampled from patients are a subset of the bacterial population, representing strains that had the opportunity to infect and survive new selective pressures associated with a pathogenic lifestyle.

The common gastrointestinal pathogen *Campylobacter jejuni* is widely distributed among wild and domesticated animal species/reservoirs (Sheppard et al. 2011), and the majority of the human infections are the result of consumption of contaminated food (Kapperud et al. 2003; Friedman et al. 2004; Skarp et al. 2016). *Campylobacter jejuni* populations are generally structured by host source (Sheppard et al. 2010, 2011), and this has allowed the attribution of the source of human infection based upon comparative multilocus sequence typing (MLST) and whole-genome characterization of host and clinical isolates (Sheppard, Dallas, MacRae, et al. 2009; Sheppard, Dallas, Strachan, et al. 2009; Pascoe et al. 2015; Dearlove et al. 2016; Thepault et al. 2017). These studies revealed chickens as a major source of human campylobacteriosis (EFSA 2015). On the assumption that all strains are equally able to infect humans, the abundance of *C. jejuni* in farmed chickens (Vidal et al. 2016) and contamination of retail poultry (Wimalarathna et al. 2013) would be enough to explain the importance of chickens as a pathogen reservoir. However, recent studies of *C. jejuni* in poultry have shown that some common chicken-associated strains are rare among clinical isolates while others increase in relative frequency (Yahara et al. 2017). This suggests that factors other than simple opportunity for transmission are involved in human infection.

In some species, such as *Escherichia coli*, the emergence of pathogenic strains can be associated with the acquisition of specific attributes which confer increased ability to cause disease or evade treatment. For example, genetic elements that encode virulence and persistence in humans such as those carried by phages and plasmids in *E. coli* or the acquisition of antibiotic resistance in *Staphylococcus*(as reviewed in Kaper et al. 2004; Pantosti et al. 2007). In some cases the acquisition of small amount of genetic material increases the virulence, as seen in the large scale outbreak of the Shiga-like-toxin producing *E. coli* O104:H4 (Frank et al. 2011). Where specific pathogenicity elements can be identified, it is relatively simple to identify the agent causing an outbreak and its molecular cause. However, in *C. jejnui*, traits associated with clinical isolates not only reflect virulence but also those that confer a fitness advantage against the various selective pressures encountered in the poultry processing chain, such as survival in the nonhost environment (Yahara et al. 2017).

The increasing availability of whole-genome data provides opportunities to investigate the genomic differences underlying variation in proteins and their motifs that may promote the proliferation of particular pathogenic strains. Epidemiological studies of *C. jejuni* from clinical samples and animal reservoirs typically reveal genetically diverse populations. However, isolates belonging to CC21 and CC45 are regularly the most common lineages isolated from human disease (Kärenlampi et al. 2007; Levesque et al. 2008; Mullner et al. 2009; Sheppard, Dallas, MacRae, et al. 2009; Sheppard, Dallas, Strachan, et al. 2009; Sanad et al. 2011; Mughini Gras et al. 2012; Sahin et al. 2012; Guyard-Nicodeme et al. 2015). Both of these lineages have been isolated from a variety of sources, including ruminants, poultry, wild birds, domesticated companion animals, as well as environmental samples (Sopwith et al. 2008; Sheppard et al. 2011, 2014). This ecological generalism may reflect a degree of genotypic and phenotypic plasticity that facilitates rapid host adaptation in a multihost environment (Read et al. 2013; Woodcock et al. 2017; Pascoe et al. 2017) but little is known about the specific genomic variations that promote proliferation of particular STs, within generalist lineages, in different niches such as human hosts.

Here we combine nucleotide-based phylogenetic analysis with amino acid sequence-based clustering to characterize populations of *C. jejuni* from humans and agricultural animals, and identify candidate genes involved in these possible host associations. Our hypothesis was that a combined methodological approach would identify subtle host-associated differences between isolates from major generalist groups. These analyses identified sublineages of the ST-21 complex that were overrepresented among isolates sampled from human disease. The putative functions of genes within human-only amino acid clusters included those important in human pathogenesis, such as flagella and capsule synthesis. Our study provides a new way of interrogating genomic data sets to identify candidate genes in a subset of strains that may indicate a population bottleneck associated with human colonization.

## Materials and Methods

### Bacterial Genomes

A total of 601 *C. jejuni* genomes were used in this analysis, previously published in various studies (Cody et al. 2013; Sheppard, Didelot, Jolley, et al. 2013; Sheppard, Didelot, Meric, et al. 2013; Pascoe et al. 2017; Yahara et al. 2017) (supplementary table S1, Supplementary Material online). The majority of these came from clinical isolates (*n* = 481) and the rest from agricultural sources, either poultry (*n *= 88) or cattle (*n* = 32). Most isolates were from the United Kingdom (*n* = 546/601, 90.1%). A total of 134/601 (22.3%) were from CC-45 and 467/601 (77.7%) were from CC-21-48-206 (supplementary table S1, Supplementary Material online), which have been shown to form a single sequence cluster in previous studies (Sheppard, Didelot, Meric, et al. 2013). These constituted all the sequenced genomes available to us when this study was initiated. CC21-48-206 is henceforth collectively referred to as CC21 group in this study. Sequencing was performed on Illumina platforms, and assemblies were performed with either Velvet (Zerbino and Birney 2008) or Spades (Bankevich et al. 2012). Assembled DNA sequences from various sources (supplementary table S1, Supplementary Material online) were uploaded to a web-based database based on the BIGSdb platform (Jolley and Maiden 2010) which allowed archiving, whole-genome gene-by-gene sequence alignments and prevalence analyses. In addition, the isolation source of all available CC21 group and CC45 isolate records (*n* = 17,107) from the pubMLST database (https://pubmlst.org/campylobacter/; last accessed February 07, 2018) were obtained (October 21, 2016) and analyzed to quantify the numbers of different STs isolated from humans and agricultural animals and contextualize this study.

### Phylogenetic Tree Inference

Sequence alignments were obtained using a gene-by-gene approach (Sheppard et al. 2012). Briefly, the presence of 1,668 coding sequences (CDS) from the reference *C. jejuni* NCTC11168 genome (NCBI accession: NC_002163.1) in all 601 genomes of this study was inferred using BLAST with the following parameters: A gene was considered present when a local alignment match with the reference was obtained on >50% of the sequence length with >70% sequence identity. Using these criteria, 1,058 genes were shared by all 601 genomes from our data set, constituting the “core genome.” Gene-by-gene alignments using MAFFT (Katoh and Standley 2013) were concatenated to create a core genome gene-by-gene alignment that was used subsequently. For protein trees, in-frame translation was performed using custom scripts (supplementary file 1, Supplementary Material online) for each individual gene alignment, which were then concatenated. The resulting concatenations were used as an input for the reconstruction of phylogenetic trees, either using an approximation of the maximum-likelihood algorithm implemented in FastTree2 (Price et al. 2010) (fig. 2) or RAxML (Stamatakis 2014) (supplementary fig. S1, Supplementary Material online). For the comparison of nucleotide and in-frame translated phylogenetic trees, we used RAxML (Stamatakis 2014) with GTRGAMMA and PROTGAMMAGTR models, respectively. For amino acid trees, the analysis used a simple search under the GAMMA model of rate heterogeneity on the protein data set using empirical base frequencies and estimating a general time reversible model of amino acid substitution.

### KPAX2 Method: Bayesian Clustering Based on Amino Acid Sequence

KPAX2 is a new Bayesian method for identifying evolutionary signals in amino acid sequences that relate to differential evolution of lineages that may be either monophyletic or polyphyletic, for example, resulting from the horizontal distribution of relevant genomic elements through recombination (Pessia et al. 2015). Earlier analysis of a database of thousands of influenza A virus H3N2 subtypes demonstrated that the method could accurately identify antigenic clusters determined by amino acid variation and the sequence positions relevant for the antigenic differences (Pessia et al. 2015). The concatenated set of 601 core genome sequences corresponded to 153,911 amino acid positions, harboring 17,405 polymorphic sites. KPAX2 was used with the default prior settings, and inference was initialized with a proposal partition of the samples obtained using the k-medoids algorithm based on Tajima and Nei (1984) pairwise distances of protein sequences together with the Tamura and Kumar (2002) correction for heterogeneous patterns. The initial number of clusters was chosen by selecting the k associated with the highest log posterior probability under the KPAX2 model. In total, 100 partitions were then created by applying random modifications to the initial partition obtained by the k-medoids solution to the proposal partition. Split, merge, and transfer operators were as previously described (Pessia et al. 2015). Each of the 100 partitions was then independently used as a starting state for the KPAX2 posterior maximization algorithm to ensure that the final estimate was as close to the global posterior mode as possible. The 100 KPAX2 runs were done in parallel on a cluster computer, where the individual runs took approximately 1–2 weeks until convergence. The clustering solution with the highest log posterior probability among the 100 independent runs was chosen as the final estimate. The source of isolates belonging to different KPAX clusters was indicated for isolates from: human clinical only (clinical); chicken and human clinical sources (chicken + clinical); cattle and human clinical sources (cattle + clinical); and chicken, cattle and human clinical sources (chicken + cattle + clinical) (supplementary table S2, Supplementary Material online). For each KPAX cluster, characteristic amino acids were determined (Pessia et al. 2015), as well as corresponding proteins and genes in the *C. jejuni* NCTC11168 reference genome (supplementary table S3, Supplementary Material online). This allowed for a comparison of KPAX clustering results with genome-wide association study (GWAS) results to identify the genes associated with clinical-only *C. jejuni* KPAX groups.

### Prevalence of STs from Human-Only KPAX Clusters among Isolates from Human and Nonhuman Sources

Total prevalence of *C. jejuni* STs observed to belong to human-only KPAX clusters was quantified among samples isolated from human and nonhuman sources (mainly poultry and cattle) and was inferred using isolation source information specified in a total of 17,107 CC21, CC48, CC206, and CC45 isolate records, taken from a total of 49,598 archived isolate records from every CC publicly available in the pubMLST database (https://pubmlst.org/campylobacter/; accessed October 21, 2016).

### SEER Method: Genome-Wide Association Mapping

We used a k-mer enrichment method to identify, from the nucleotide sequence data, which genomic elements were significantly more prevalent in two groups of isolates: The human-only KPAX clusters (group 1, *n* = 103) compared to the remainder of the *C. jejuni* population (group 2, *n* = 498) (Weinert et al. 2015; Lees et al. 2016). This binary trait analysis was performed to ensure that eventual gene regulatory elements or accessory genes associated with the clusters would not remain unidentified, because the KPAX2 method is based only on core protein sequence variation. The input assemblies contained approximately 31 M unique k-mers with lengths between 10 and 99 nucleotides. The following filtering steps were applied to reduce the original k-mer input set by including only k-mers that: 1) had >75% frequency in group 1 and <25% frequency in group 2; 2) had a chi-square association test *P*-value < 10^−8^; and 3) had association *P*-value < 10^−8^ in a logistic regression model with the three first multidimensional scaling coordinates representing the population structure correction. The multidimensional scaling coordinates were calculated from a distance matrix based on 10,000 randomly selected k-mers from the initial set. The final set of genome-wide significant k-mers contained 347 k-mers, which were mapped to an annotated reference genome to identify their contexts.

## Results

### STs Vary in Frequency in Human Clinical and Agricultural Environments

**Fig. 1.**
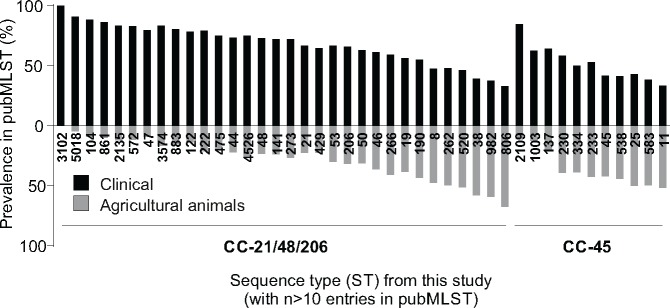
—Prevalence of clinical and agricultural *C. jejuni* within ST-21 and ST-45 CCs in a public archive repository. The prevalence of clinical (black) and poultry/livestock (gray) isolation sources in pubMLST for each ST in our data set with more than ten isolate records in the pubMLST database (https://pubmlst.org/campylobacter/; last accessed February 07, 2018). There were a total of 17,107 archived public isolate records.

Direct comparison of the relative prevalence of sequence types was performed using the entire *Campylobacter* PubMLST database. This contained a total of 49,598 entries on October 21, 2016. Of these 13,095 belonged to the CCs 21, 48, and 206, previously shown to form a single sequence cluster based upon whole-genome analysis, and 4,012 belonged to CC45 complex. Within the CC21 group there were 8,382 human clinical isolates and 3,869 originating from agricultural animal sources, while in CC45 there were 1,674 human clinical isolates and 1,685 agricultural isolates. The relative abundance of isolate STs belonging to CC21-48-206 and CC45 was determined (fig. 1). In both CCs, there was variation in the relative frequency of STs isolated from human clinical and agricultural animal samples.

### Amino Acid Sequence-Based Analysis Reveals Human-Only Subclusters

**Fig. 2.**
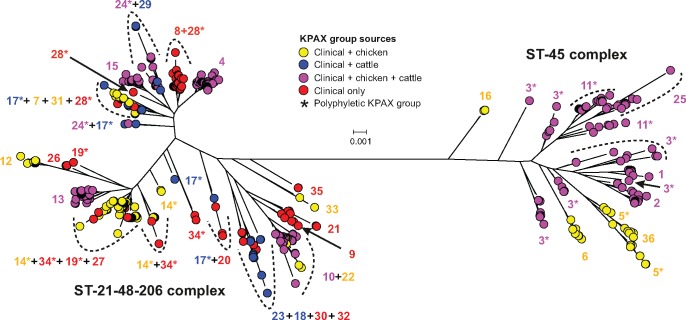
—Population structure of 601 *C. jejuni* ST-21 and ST-45 complex isolates. Isolates are labeled by KPAX group labels (integers) and colored by their source distribution within KPAX groups: Isolates from chicken and clinical sources (yellow), cattle and clinical sources (blue), chicken, cattle and clinical sources (pink), or clinical only (red). Polyphyletic KPAX groups, reflecting isolates in the same KPAX group but in multiple lineages on the tree, are indicated with an asterisk. The phylogenetic tree was reconstructed from a whole-genome gene-by-gene amino acid alignment, translated in-frame, using an approximation of the maximum-likelihood algorithm implemented in FastTree2, and using a general time reversible model.

The Bayesian model-based method KPAX2 was used to classify aligned proteins into functionally divergent groups, based upon amino acid residues of a collection of 601 genomes representing 66 STs belonging to the CC21 group and CC45. A total of 1,058 core CDS used in the nucleotide phylogeny were in silico translated and a concatenated amino acid alignment produced for each genome-sequenced strain. We then performed Bayesian clustering using the KPAX2 algorithm, and the tree was annotated with the 36 KPAX clusters identified (fig. 2). KPAX groups could be classified into four categories depending on sources of isolates: Human only (12 KPAX groups, 112 isolates from 20 STs), human and chicken only (10 KPAX groups, 150 isolates from 20 STs), human and cattle only (4 KPAX groups, 33 isolates from 13 STs), and human, chicken and cattle (10 KPAX groups, 306 isolates from 24 STs). The isolate source within each KPAX group is shown in the supplementary table S2, Supplementary Material online.

KPAX and nucleotide sequence clusters showed incomplete congruence. Amino acid clustering was polyphyletic when superimposed on the nucleotide phylogeny (fig. 2, supplementary fig. S1, Supplementary Material online) and in some cases, divergent lineages shared the same KPAX cluster. For example, the 138 isolates belonging to ST-21 were found in 7 different KPAX groups containing isolates from various sources. However, particular STs (ST-21, ST-50, ST-47, ST-44, ST-861, and ST-190) were assigned KPAX groups encompassing only isolates from humans. Examination of isolate records in the entire pubMLST database revealed that most isolates from STs assigned to human-only KPAX groups (276/283 isolates, in 15/20 STs) have also been isolated from humans and other host species, with only ST-6601, ST-6137, ST-5727, and ST-2355 having been isolated solely from humans (table 1). Obviously, KPAX clusters were not defined using the whole genomes of the pubMLST-archived comparative data set; however, it is useful to contextualize KPAX-ST correlation within a wider data set. It should be noted that the ST designation can have poor specificity in contrast to the lineages determined from whole genomes and therefore an isolate from a nonhuman host present in the pubMLST database may lack the genetic elements identified in our present analysis.

**Table 1:** Prevalence of isolates from STs found in human-only KPAX groups in human and nonhuman sources

KPAX Group	ST	Total Number of Isolates in Our Study	Associated Hosts	**Prevalence in Human Hosts in pubMLST (%)**^a^	**Prevalence in Nonhuman Hosts in pubMLST (%)**^a^
KPAX-8	ST-21*	138	Human, chicken, cattle	66.5	22.4
KPAX-9	ST-475	5	Human	75.0	19.4
	ST-6601#	1	Human	100.0	0.0
KPAX-19	ST-50*	100	Human, chicken	62.8	31.4
	ST-5727#	2	Human	100.0	0.0
	ST-2355#	1	Human	100.0	0.0
KPAX-20	ST-47*	3	Human	79.2	9.4
	ST-5242#	1	Human	100.0	0.0
KPAX-21	ST-572	4	Human	82.7	11.8
	ST-5138	1	Human	66.7	33.3
KPAX-26	ST-44*	6	Human	73.2	22.3
KPAX-27	ST-50*	100	Human, chicken	62.8	31.4
KPAX-28	ST-21*	138	Human, chicken, cattle	66.5	22.4
	ST-861*	4	Human	86.2	10.3
	ST-5018	3	Human	90.5	4.8
	ST-190*	2	Human	54.7	43.1
	ST-141	1	Human	72.0	24.0
KPAX-30	ST-222	3	Human	78.9	21.1
KPAX-32	ST-122	4	Human	78.2	13.9
KPAX-34	ST-21*	138	Human, chicken, cattle	66.5	22.4
	ST-50*	100	Human, chicken	62.8	31.4
	ST-3769	1	Human	83.3	16.7
	ST-520	1	Human	46.1	51.3
KPAX-35	ST-6137#	2	Human	100.0	0.0

Note.— Asterisks indicate STs that also found in other nonhuman-only KPAX groups. Dashes indicate STs that have never been isolated from nonhuman sources in our data set or pubMLST.

^a^ pubMLST (https://pubmlst.org/campylobacter/) as accessed on October 21, 2016.

### Identification of Genes with Human-Associated Amino Acid Signatures within the CC21 Group

**Fig. 3.**
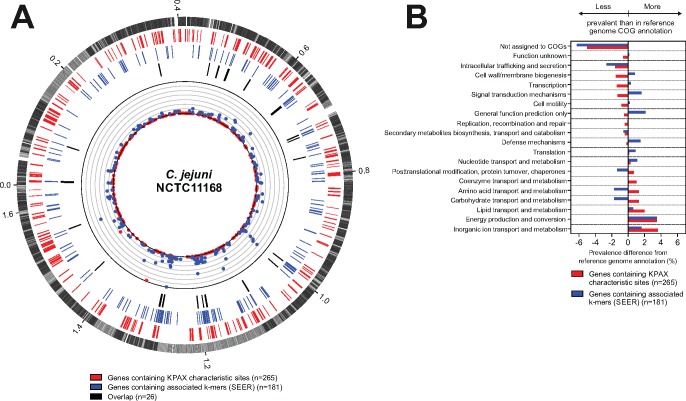
—Genes associated with clinical-only *C. jejuni* KPAX groups. (*A*) GWAS results visualized on a circular reference genome. The outer circle indicates genes from the *C. jejuni* NCTC1168 reference genome, with core genes shared by all isolates in our data set (black) and accessory genes (gray) indicated. Genes found to contain characteristic amino acid sites defining KPAX groups are represented (red ticks) along with a quantitative visualization of the number of these sites per gene (red dots; scale of the quantification from 0 to 420). Genes found to contain k-mers associated with clinical-only KPAX groups using SEER are represented (blue ticks) along with a quantitative visualization of the number of these k-mers mapped per gene (blue dots; scale of the quantification from 0 to 25). Black ticks indicate genes containing both KPAX group characteristic sites and associated k-mers using SEER. (*B*) Difference in COGs prevalence (%) among genes containing KPAX characteristic sites (red) and genes containing associated k-mers inferred by SEER (blue) with COGs prevalence in the *C. jejuni* NCTC11168 reference genome annotation.

We sought to identify the discriminatory amino acids that resulted in clustering of human clinical-only CC21 group isolates. We identified a total of 1,213 amino acids sites which mapped to 265 genes (supplementary table S4, Supplementary Material online). Mapping the physical location of these against the reference CC21 genome NCTC11168 suggested that these loci were distributed across the genome and not under strong linkage disequilibrium resulting from physical proximity (fig. 3*A*). Interestingly, a total of 24/265 (9.0%) genes were found to be associated with previous GWASs (supplementary table S4, Supplementary Material online). More specifically, 3 genes were predicted to have a role in survival from farm to clinical disease (Yahara et al. 2017), 8 genes to have a role in in vitro colonization of surfaces and aggregation (Pascoe et al. 2015), and 14 genes to have a role in nonhuman host adaptation (Sheppard, Didelot, Meric, et al. 2013) (supplementary table S4, Supplementary Material online). Although some of these associations were sometimes weak in the corresponding studies, they were nonetheless highlighted and are consistent with a general role in transmission and host colonization.

To confirm whether these loci were associated with a human clinical-only sublineage we also performed sequence element enrichment analysis, using SEER (Lees et al. 2016), to identify the genetic basis of human clinical-only sublineage strains compared with those from other host sources (fig. 3, supplementary tables S5 and S6, Supplementary Material online). A total of 181 genes (supplementary table S5, Supplementary Material online), containing 547 enriched k-mers, were obtained (supplementary table S6, Supplementary Material online). These included genes that have been identified in previous association studies (supplementary table S5, Supplementary Material online), in particular genes with putative roles in in vitro colonization of surfaces and aggregation, host adaptation and clinical disease (Sheppard, Didelot, Meric, et al. 2013; Pascoe et al. 2015; Yahara et al. 2017).

A total of 26 genes were significantly associated with human-only lineages in both KPAX clustering and SEER association analyses (fig. 3, table 2). Half of these genes have been described as important for host colonization or pathogenesis, nine in humans or human cell studies, and four in chicken colonization studies (table 2), consistent with a broad role for these genes in host adaptation and/or in multihost fitness. Of particular note within these genes were the flagellar gene *flgH* highlighted in a previous GWAS on nonchicken host adaptation (Sheppard, Didelot, Meric, et al. 2013), two genes (*ceuC* and *ceuE*) involved in the enterochelin iron uptake system in *C. jejuni*, a gene (*aspB*) involved in aspartate metabolism, and a gene (*fdhD*) encoding a formate dehydrogenase, a function that has been highlighted as important for survival from farm to clinical disease (Yahara et al. 2017). All five of these genes are known to be important in the invasion of mammalian cells and/or human colonization (Palyada et al. 2004; Guerry 2007; Novik et al. 2010; Sheppard, Didelot, Meric, et al. 2013; Yahara et al. 2017).

**Table 2:** List of Genes Associated with Clinical-Only *Campylobacter jejuni* KPAX Groups

Name	Alias	Operon^a^	Predicted Product (COG)	COG Code	COG Description	Number of Characteristic Sites (KPAX)	Number of Mapping k-mers (SEER)	Notes	References
*cj1346c*	*dxr*	500	1-Deoxy-d-xylulose 5-phosphate reductoisomerase	I	Lipid transport and metabolism genes	52	8		
*cj1347c*	*cdsA*	500	Phosphatidate cytidylyltransferase	I	Lipid transport and metabolism genes	8	1	maf adhesins are included in the maf6-Cj1347 genomic region	(46)
*cj1253*	*pnp*	472	Polynucleotide phosphorylase/polyadenylase	J	Translation	7	5		
*cj0762c*	*aspB*	285	Aspartate aminotransferase	E	Amino acid transport and metabolism genes	6	1	A *aspB* mutant is defective for entry into cultured human epithelial cells	(38)
*cj0810*	*nadE*	301	NAD synthetase	H	Coenzyme transport and metabolism genes	6	1		
*cj0006*	*—*	4	Putative Na+/H+ antiporter family protein	R	General function prediction only	5	4	Cj0006 is expressed in vivo when *C. jejuni* infects chicken	(48)
*cj0389*	*serS*	149	Seryl-tRNA synthetase	J	Translation	5	1		
*cj0542*	*hemA*	213	Glutamyl-tRNA reductase	H	Coenzyme transport and metabolism genes	3	3		
*cj0767c*	*coaD*	286	Phosphopantetheine adenylyltransferase	H	Coenzyme transport and metabolism genes	3	1		
*cj1620c*	*mutY*	593	A/G-specific adenine glycosylase	L	Replication, recombination and repair	3	2	An SNP in mutY is associated with increase of antibiotic resistance	Dai et al. (2015).
*cj0005c*	*—*	3	Molydopterin containing oxidoreductase	R	General function prediction only	2	2	Infection of and adherence to human Caco2 cells in vitro was strongly reduced in a cj0005c mutant	(47)
*cj0069*	*—*	38	Hypothetical protein Cj0069	J	Translation	2	1	Involved in the proximal response to cell adhesion and biofilm formation	Asakura et al. (2007).
*cj0598*	*—*	231	Hypothetical protein Cj0598	S	Function unknown genes	2	5		
*cj0689*	*ackA*	259	Acetate kinase	C	Energy production and conversion genes	2	2	Involved in nutrient acquisition, acetate metabolism	
*cj1076*	*proC*	404	Pyrroline-5-carboxylate reductase	E	Amino acid transport and metabolism genes	2	1		
*cj1157*	*dnaX*	426	DNA polymerase III subunits gamma and tau	L	Replication, recombination and repair	2	2	Highlighted in a study as a putative Guillain–Barre syndrome marker	(52)
*cj1508c*	*fdhD*	555	Formate dehydrogenase accessory protein	C	Energy production and conversion genes	2	3	Formate metabolism is involved in host association and survival in the food chain from farm to human disease	(12)
*cj0498*	*trpC*	200	Indole-3-glycerol-phosphate synthase	E	Amino acid transport and metabolism genes	1	2	In a genomic region identified as important for cell hyperinvasiveness in a transposon assay	(53)
*cj0518*	*htpG*	206	Heat shock protein 90	O	Posttranslational modification, protein turnover, chaperones genes	1	1	Associated in GWAS on biofilm formation (heatshock protein); Pascoe et al. (2017)	
*cj0543*	*proS*	213	Prolyl-tRNA synthetase	J	Translation	1	3		
*cj0687c*	*flgH*	258	Flagellar basal body L-ring protein	N	Cell motility genes	1	3	Flagellar assembly cluster; flagellar motility is important for human and chicken colonization, and possible secretion of virulence factors/Associated with cattle adaptation in GWAS	(23, 37)
*cj1056c*	*—*	398	Putative carbon–nitrogen hydrolase family protein	R	General function prediction only	1	1	Expression of cj1056c is modulated at low pH in vitro	Reid et al. (2008).
*cj1261*	*racR*	477	Two-component regulator	K	Transcription	1	6	The *Campylobacter* RacRS system regulates fumarate utilization in a low oxygen environment, and racR mutants show reduced colonization of chicken	(50, 51)
*cj1271c*	*tyrS*	479	Tyrosyl-tRNA synthetase	J	Translation	1	1	TyrS was overexpressed in a poor colonizer of chicken/Associated with cattle adaptation in GWAS	(23, 49)
*cj1353*	*ceuC*	502	Enterochelin uptake permease	P	Inorganic ion transport and metabolism genes	1	5	Uptake of siderophores is a described virulence/host colonization trait	(45)
*cj1355*	*ceuE*	502	Enterochelin uptake periplasmic binding protein	P	Inorganic ion transport and metabolism genes	1	5	ceuE mutant shows decreased chicken colonization	(39)

Note.—Genes are overlapping between the two analyses (KPAX and SEER).

^a^ As predicted by OperonPredictor (http://biocomputo2.ibt.unam.mx/OperonPredictor/; last accessed February 07, 2018).

## Discussion

An important aim in zoonotic pathogen research is to identify genetic and functional variations associated with lineages or sublineages that cause human infection. Comparative analysis of nucleotide sequence variation across the genome has improved understanding of the epidemiology and evolution of *Campylobacter* (Sheppard, Didelot, Jolley, et al. 2013; Gilbert et al. 2016; Llarena et al. 2016). Although this has provided a basis for identifying candidate genes with potential functional significance (Morley et al. 2015; Pascoe et al. 2015; Yahara et al. 2017), straight forward genome analysis often ignores factors relating translation and the production of specific amino acid chains and proteins that may be important in host adaptation or pathogenicity. For example, although the four nucleotides can form 64 different triplets they only encode 20 amino acids. This means that the same amino acid can be encoded by different triplets, typically with variation at the third base, and divergent genomes may have convergent amino acid sequences that are potentially functionally important in host adaptation or pathogenesis. Analysis of encoded amino acid sequences in this study identified polyphyletic nucleotide sequence clusters within the CC21 group that clustered together within the same amino acid sequence clusters. These convergent human-only amino acid KPAX clusters, in divergent genomic backgrounds, may have been overlooked using conventional nucleotide sequence-based approaches.

Comparative analysis of the nucleotide sequence of the 601 *C. jejuni* genomes in this study identified STs belonging to the CC21 group and CC45 that were reported to have been isolated at different frequencies from agricultural animal and human sources lineages. This is consistent with other population genomic studies, where the variation in relative abundance has been explained by the different capacity of certain strains to survive through the poultry production chain at atmospheric oxygen concentrations (Yahara et al. 2017). Asymptomatic carriage of *C. jejuni* is not thought to be common in humans in industrialized countries (Lee et al. 2013). Therefore, under a simple transmission model, amino acid clusters would be expected to be present in both reservoir animal and infected human hosts. For this reason, the existence of strongly human-only amino acid KPAX clusters is unexpected. There are two possible explanations. First, isolates assigned to human-only KPAX clusters are derived from a source that is not represented in our isolate collection, which has not been captured by the sampling of isolates used in this study. Second, there are isolates that share amino acid clusters within CC21 group *C. jejuni* in our data set that increase in relative frequency in humans, compared with the isolates from other hosts. Additionally, it is possible that asymptomatic carriage of *Campylobacter* may be underestimated and underreported (Calva et al. 1988; Louwen et al. 2012; Lee et al. 2013; Islam et al. 2017). These factors could influence the evolution and population structure of symptomatic bacteria.

Examination of isolate records in the entire pubMLST database revealed that 97% of the isolates assigned to human-only amino acid KPAX clusters are of STs that have been isolated from other host species as well as humans (table 1). Notably, only five STs from human-only KPAX groups (corresponding to 7/276 isolates in our data set) have never been reported in nonhuman hosts, either in our data set or from isolate records in pubMLST. On the basis of the known sources of *C. jejuni* in human infection—including CC21 group isolates (Sheppard, Dallas, MacRae, et al. 2009; Sheppard, Dallas, Strachan, et al. 2009), the close similarity between *C. jejuni* populations on food and those from clinical samples (Kittl et al. 2013), and the presence of STs belonging to human-only amino acid KPAX clusters among agricultural hosts in pubMLST, it is unlikely that they indicate an unknown host source population, although this cannot be ruled out in this study.

Our results are therefore consistent with the increase in relative frequency of particular amino acid sequence subclusters that are uncommon in animal hosts, among isolates from humans. Host colonization potential is influenced by the adaptive genomic variations that exist before and after transmission to the new host species (Geoghegan et al. 2016). In both cases, population bottlenecks reduce the genetic variance in the population at interhost transmission which would account for the increased relative frequency of human-only amino acid KPAX clusters. It remains difficult to differentiate genetic changes associated with bottlenecking and drift from adaptive physiological changes that directly impact pathogenesis, such as human tissue tropism and virulence. Furthermore, human passage can induce genetic variation in contingency genes coding surface structure through frame shifts and phase variation (Bayliss et al. 2012; Revez et al. 2013; Thomas et al. 2014). However, the sharing of amino acid sequence clusters by polyphyletic lineages is evidence of homoplasy and investigating the putative function of these genes may provide clues about their potential role in human colonization. Human-only KPAX clusters are present in every major lineage within the CC21 group (fig. 2) and are notably absent among CC45 isolates. This asymmetry cannot be explained by an insufficient sample size from the CC45 population in our data set and may suggest that, despite being an efficient human colonizer, CC45 strains may lack the suitable genetic background for acquisition of genomic elements that are associated with elevated human colonization that we observe in the CC21 group. Further analysis of larger sample sets, potentially including phenotypic analyses, is needed to confirm this.

Genome-wide association methods that have recently been applied to bacteria (Sheppard, Didelot, Meric, et al. 2013) allow the investigation of genetic variation that underlies important phenotypes. By quantifying the nucleotide sequence that was enriched in isolates from humans (Lees et al. 2016) across the genomes, we were able to investigate the putative function of genes with human-only amino acid KPAX clusters. A total of 26 genes were identified (table 2), half of which have been previously linked to host colonization or pathogenesis, nine in humans or human cells, four in chicken. For example, *flgH*, a gene associated with flagellar assembly (table 2) and otherwise associated with adaptation in a mammalian host (Sheppard, Didelot, Meric, et al. 2013). Flagellar motility has been shown to be important for human and chicken colonization, and possibly for the secretion of virulence factors into host cells (Guerry 2007). Genes directly involved in host colonization also included *ceuCE*, involved in enterochelin uptake (table 2). The uptake of siderophore has been described as a virulence/host colonization trait in *Campylobacter* (Richardson and Park 1995), and a *ceuE* mutant has been shown to be altered in chicken colonization abilities (Palyada et al. 2004). Additionally, the *cdsA* gene is located in the genomic region of known *maf* adhesins, involved in survival and host colonization (Karlyshev et al. 2002). Knockout mutants of *cj0005c*, an uncharacterized oxidoreductase, have been shown to be strongly impaired in infection abilities and adherence to human Caco2 cells in vitro (Tareen et al. 2011), whereas a neighboring gene, *cj0006*, encoding a putative transporter, has been shown in global transcriptomic studies to be overexpressed in vivo when *C. jejuni* infects chicken (Hu et al. 2014). Finally, the *tyrS* gene, predicted to encode a tyrosyl-tRNA synthetase, has been observed to be overexpressed in a poor chicken colonizer strain of *C. jejuni* (Seal et al. 2007). Additionally, it has been associated with mammalian (cattle) adaptation in a previous GWAS from our laboratory (Sheppard, Didelot, Meric, et al. 2013).

Genes predicted to have a role in metabolism were also highlighted. The *ackA* and *aspB* genes are involved in acetate and aspartate metabolism, respectively, and have been shown in mutagenesis studies to be important for entry into human epithelial cells in vitro (Novik et al. 2010). The *fdhD* gene encoding a formate dehydrogenase was also associated with isolates belonging to human-only amino acid clusters. Formate metabolism has been previously implicated in host association and survival in the food production chain from farm to human disease (Yahara et al. 2017). The *racR* gene which regulates fumarate utilization in a low-oxygen environment also displayed human-associated variation and *racR*-deficient mutants have shown reduced chicken colonization in vivo (Bras et al. 1999; van der Stel et al. 2015). Other genes with variation associated with the CC21 human amino acid clusters included the *dnaX* gene that encodes a DNA polymerase and is a marker for the campylobacteriosis sequelae Guillain–Barre syndrome (Godschalk et al. 2006) and *trpC* that encodes an indole-3-glycerol-phosphate synthase in a genomic region important for human cell hyperinvasiveness (Javed et al. 2010).

Genomic variation associated with clinical *C. jejuni* isolates includes elements associated with the primary host (Sheppard, Didelot, Meric, et al. 2013) and the food production chain (Yahara et al. 2017), as well as variation which confers an adaptive advantage to human colonization and may directly impact pathogenesis (Thompson and Gaynor 2008). Evidence of genetic bottlenecks and selection fostered by this complex fitness landscape will not only be reflected in nucleotide sequence variation but also in features, such as gene order, distribution of CDS on leading and lagging strands, GC skew, and codon usage (Bentley and Parkhill 2004; Rocha 2004). By combining analysis of nucleotide sequence and amino acid variation we were able to identify a subset of human-associated *C. jejuni*. As these isolates are found in nonhuman hosts, we interpret this as evidence of a genetic bottleneck that increases the relative frequency of certain strains in the infected individuals. Although larger scale studies are necessary to confirm a potential adaptive role for the human-associated variation, our analysis has identified a group of human-pathogenic *C. jejuni* that do not exhibit typical source-sink epidemiology, potentially reflecting human tissue tropism or virulence.

## Supplementary Material

Supplementary data are available at *Genome Biology and Evolution* online.

## Supplementary Material

Supplementary Data

## References

[evy026-B601] Asakura H, Yamasaki M, Yamamoto S, Igimi S. 2007 Deletion of peb4 gene impairs cell adhesion and biofilm formation in Campylobacter jejuni. FEMS Microbiol Lett. 275 2:278–285. 17714477 10.1111/j.1574-6968.2007.00893.x

[evy026-B1] Bankevich A, 2012 SPAdes: a new genome assembly algorithm and its applications to single-cell sequencing. J Comput Biol. 19 5:455–477.http://dx.doi.org/10.1089/cmb.2012.0021 22506599 10.1089/cmb.2012.0021 PMC3342519

[evy026-B2] Bayliss CD, 2012 Phase variable genes of *Campylobacter jejuni* exhibit high mutation rates and specific mutational patterns but mutability is not the major determinant of population structure during host colonization. Nucleic Acids Res. 40 13:5876–5889.http://dx.doi.org/10.1093/nar/gks246 22434884 10.1093/nar/gks246 PMC3401435

[evy026-B3] Bentley SD, Parkhill J. 2004 Comparative genomic structure of prokaryotes. Annu Rev Genet. 38:771–792.http://dx.doi.org/10.1146/annurev.genet.38.072902.094318 15568993 10.1146/annurev.genet.38.072902.094318

[evy026-B4] Bras AM, Chatterjee S, Wren BW, Newell DG, Ketley JM. 1999 A novel *Campylobacter jejuni* two-component regulatory system important for temperature-dependent growth and colonization. J Bacteriol. 181 10:3298–3302. 10322038 10.1128/jb.181.10.3298-3302.1999 PMC93792

[evy026-B5] Calva JJ, Ruiz-Palacios GM, Lopez-Vidal AB, Ramos A, Bojalil R. 1988 Cohort study of intestinal infection with campylobacter in Mexican children. Lancet 1 8584:503–506. 2893920 10.1016/s0140-6736(88)91297-4

[evy026-B6] Cody AJ, 2013 Real-time genomic epidemiological evaluation of human *Campylobacter* isolates by use of whole-genome multilocus sequence typing. J Clin Microbiol. 51 8:2526–2534.http://dx.doi.org/10.1128/JCM.00066-13 23698529 10.1128/JCM.00066-13 PMC3719633

[evy026-B7] Dearlove BL, 2016 Rapid host switching in generalist *Campylobacter* strains erodes the signal for tracing human infections. ISME J. 10 3:721–729.http://dx.doi.org/10.1038/ismej.2015.149 26305157 10.1038/ismej.2015.149 PMC4677457

[evy026-B602] Dai L, Muraoka WT, Wu Z, Sahin O, Zhang Q. 2015 A single nucleotide change in mutY increases the emergence of antibiotic-resistant Campylobacter jejuni mutants. J Antimicrob Chemother. 70 10:2739–2748. 26169557 10.1093/jac/dkv190 PMC4668879

[evy026-B8] EFSA 2015. The European Union summary report on trends and sources of zoonoses, zoonotic agents and food-borne outbreaks in 2013. EFSA J. 13(1):3991. 10.2903/j.efsa.2018.5500 PMC7009540 32625785

[evy026-B9] Frank C, 2011 Epidemic profile of Shiga-toxin-producing *Escherichia coli* O104:H4 outbreak in Germany. N Engl J Med. 365 19:1771–1780. 21696328 10.1056/NEJMoa1106483

[evy026-B10] Friedman CR, ; Emerging Infections Program FoodNet Working Group. 2004 Risk factors for sporadic *Campylobacter* infection in the United States: a case-control study in FoodNet sites. Clin Infect Dis. 38(s3):S285–S296. 15095201 10.1086/381598

[evy026-B11] Geoghegan JL, Senior AM, Holmes EC. 2016 Pathogen population bottlenecks and adaptive landscapes: overcoming the barriers to disease emergence. Proc Biol Sci. 283 1837:20160727. 27581875 10.1098/rspb.2016.0727 PMC5013787

[evy026-B12] Gilbert MJ, 2016 Comparative genomics of *Campylobacter* fetus from reptiles and mammals reveals divergent evolution in host-associated lineages. Genome Biol Evol. 8 6:2006–2019. 27333878 10.1093/gbe/evw146 PMC4943207

[evy026-B13] Godschalk PC, 2006 Identification of DNA sequence variation in *Campylobacter jejuni* strains associated with the Guillain-Barre syndrome by high-throughput AFLP analysis. BMC Microbiol. 6:32. 16594990 10.1186/1471-2180-6-32 PMC1513382

[evy026-B14] Guerry P. 2007 *Campylobacter flagella*: not just for motility. Trends Microbiol. 15 10:456–461.http://dx.doi.org/10.1016/j.tim.2007.09.006 17920274 10.1016/j.tim.2007.09.006

[evy026-B15] Guyard-Nicodeme M, 2015 Prevalence and characterization of *Campylobacter jejuni* from chicken meat sold in French retail outlets. Int J Food Microbiol. 203:8–14.http://dx.doi.org/10.1016/j.ijfoodmicro.2015.02.013 25770428 10.1016/j.ijfoodmicro.2015.02.013

[evy026-B16] Hu Y, Huang J, Jiao XA. 2014 Screening of genes expressed in vivo during interaction between chicken and *Campylobacter jejuni*. J Microbiol Biotechnol. 24 2:217–224.http://dx.doi.org/10.4014/jmb.1308.08092 24225374 10.4014/jmb.1308.08092

[evy026-B17] Islam Z, 2017 Capsular genotype and lipooligosaccharide locus class distribution in *Campylobacter jejuni* from young children with diarrhea and asymptomatic carriers in Bangladesh. Eur J Clin Microbiol Infect Dis. doi:10.1007/s10096-017-3165-7. 10.1007/s10096-017-3165-7 29270862

[evy026-B18] Javed MA, 2010 Transposon mutagenesis in a hyper-invasive clinical isolate of *Campylobacter jejuni* reveals a number of genes with potential roles in invasion. Microbiology 156 4:1134–1143.http://dx.doi.org/10.1099/mic.0.033399-0 20035004 10.1099/mic.0.033399-0

[evy026-B19] Jolley KA, Maiden MC. 2010 BIGSdb: scalable analysis of bacterial genome variation at the population level. BMC Bioinformatics 11:595. 21143983 10.1186/1471-2105-11-595 PMC3004885

[evy026-B20] Kaper JB, Nataro JP, Mobley HL. 2004 Pathogenic *Escherichia coli*. Nat Rev Microbiol. 2 2:123–140.http://dx.doi.org/10.1038/nrmicro818 15040260 10.1038/nrmicro818

[evy026-B21] Kapperud G, 2003 Factors associated with increased and decreased risk of *Campylobacter* infection: a prospective case-control study in Norway. Am J Epidemiol. 158 3:234–242. 12882945 10.1093/aje/kwg139

[evy026-B22] Kärenlampi R, Rautelin H, Schönberg-Norio D, Paulin L, Hänninen M-L. 2007 Longitudinal study of Finnish *Campylobacter jejuni* and *C. coli* isolates from humans, using multilocus sequence typing, including comparison with epidemiological data and isolates from poultry and cattle. Appl Environ Microbiol. 73 1:148–155. 17085689 10.1128/AEM.01488-06 PMC1797135

[evy026-B23] Karlyshev AV, Linton D, Gregson NA, Wren BW. 2002 A novel paralogous gene family involved in phase-variable flagella-mediated motility in *Campylobacter jejuni*. Microbiology 148(Pt 2):473–480. 11832511 10.1099/00221287-148-2-473

[evy026-B24] Katoh K, Standley DM. 2013 MAFFT multiple sequence alignment software version 7: improvements in performance and usability. Mol Biol Evol. 30 4:772–780.http://dx.doi.org/10.1093/molbev/mst010 23329690 10.1093/molbev/mst010 PMC3603318

[evy026-B25] Kittl S, 2013 Comparison of genotypes and antibiotic resistances of *Campylobacter jejuni* and *Campylobacter coli* on chicken retail meat and at slaughter. Appl Environ Microbiol. 79 12:3875–3878.http://dx.doi.org/10.1128/AEM.00493-13 23584778 10.1128/AEM.00493-13 PMC3675953

[evy026-B26] Lee G, 2013 Symptomatic and asymptomatic *Campylobacter* infections associated with reduced growth in Peruvian children. PLoS Negl Trop Dis. 7 1:e2036. 23383356 10.1371/journal.pntd.0002036 PMC3561130

[evy026-B27] Lees JA, 2016 Sequence element enrichment analysis to determine the genetic basis of bacterial phenotypes. Nat Commun. 7:12797. 27633831 10.1038/ncomms12797 PMC5028413

[evy026-B28] Levesque S, Frost E, Arbeit RD, Michaud S. 2008 Multilocus sequence typing of *Campylobacter jejuni* isolates from humans, chickens, raw milk, and environmental water in Quebec, Canada. J Clin Microbiol. 46 10:3404–3411.http://dx.doi.org/10.1128/JCM.00042-08 18701662 10.1128/JCM.00042-08 PMC2566118

[evy026-B29] Llarena AK, 2016 Monomorphic genotypes within a generalist lineage of *Campylobacter jejuni* show signs of global dispersion. Microb Genom. 2 10:e000088. 28348829 10.1099/mgen.0.000088 PMC5359405

[evy026-B30] Louwen R, 2012 *Campylobacter* bacteremia: a rare and under-reported event? Eur J Microbiol Immunol (Bp). 2 1:76–87. 24611124 10.1556/EuJMI.2.2012.1.11 PMC3933993

[evy026-B31] Morley L, 2015 Gene loss and lineage-specific restriction-modification systems associated with niche differentiation in the *Campylobacter jejuni* sequence type 403 clonal complex. Appl Environ Microbiol. 81 11:3641–3647.http://dx.doi.org/10.1128/AEM.00546-15 25795671 10.1128/AEM.00546-15 PMC4421040

[evy026-B32] Mughini Gras L, 2012 Risk factors for campylobacteriosis of chicken, ruminant, and environmental origin: a combined case-control and source attribution analysis. PLoS One 7 8:e42599. 22880049 10.1371/journal.pone.0042599 PMC3411806

[evy026-B33] Mullner P, 2009 Assigning the source of human campylobacteriosis in New Zealand: a comparative genetic and epidemiological approach. Infect Genet Evol. 9 6:1311–1319.http://dx.doi.org/10.1016/j.meegid.2009.09.003 19778636 10.1016/j.meegid.2009.09.003

[evy026-B34] Novik V, Hofreuter D, Galan JE. 2010 Identification of *Campylobacter jejuni* genes involved in its interaction with epithelial cells. Infect Immun. 78 8:3540–3553.http://dx.doi.org/10.1128/IAI.00109-10 20515930 10.1128/IAI.00109-10 PMC2916286

[evy026-B35] Palyada K, Threadgill D, Stintzi A. 2004 Iron acquisition and regulation in *Campylobacter jejuni*. J Bacteriol. 186 14:4714–4729. 15231804 10.1128/JB.186.14.4714-4729.2004 PMC438614

[evy026-B36] Pantosti A, Sanchini A, Monaco M. 2007 Mechanisms of antibiotic resistance in *Staphylococcus aureus*. Future Microbiol. 2 3:323–334.http://dx.doi.org/10.2217/17460913.2.3.323 17661706 10.2217/17460913.2.3.323

[evy026-B37] Pascoe B, 2015 Enhanced biofilm formation and multi-host transmission evolve from divergent genetic backgrounds in *Campylobacter jejuni*. Environ Microbiol. 17 11:4779–4789.http://dx.doi.org/10.1111/1462-2920.13051 26373338 10.1111/1462-2920.13051 PMC4862030

[evy026-B38] Pascoe B, 2017 Local genes for local bacteria: evidence of allopatry in the genomes of transatlantic *Campylobacter* populations. Mol Ecol. 26 17:4497–4508. 28493321 10.1111/mec.14176 PMC5600125

[evy026-B39] Pessia A, Grad Y, Cobey S, Puranen JS, Corander J. 2015 K-Pax2: Bayesian identification of cluster-defining amino acid positions in large sequence datasets. Microb Genom. 1 1:e000025. 28348810 10.1099/mgen.0.000025 PMC5320600

[evy026-B40] Price MN, Dehal PS, Arkin AP. 2010 FastTree 2–approximately maximum-likelihood trees for large alignments. PLoS One 5 3:e9490. 20224823 10.1371/journal.pone.0009490 PMC2835736

[evy026-B41] Read DS, 2013 Evidence for phenotypic plasticity among multihost *Campylobacter jejuni* and *C. coli* lineages, obtained using ribosomal multilocus sequence typing and Raman spectroscopy. Appl Environ Microbiol. 79 3:965–973.http://dx.doi.org/10.1128/AEM.02521-12 23204423 10.1128/AEM.02521-12 PMC3568554

[evy026-B603] Reid AN, 2008 Identification of Campylobacter jejuni genes contributing to acid adaptation by transcriptional profiling and genome-wide mutagenesis. Appl Environ Microbiol. 74 5:1598–1612. 18192408 10.1128/AEM.01508-07 PMC2258640

[evy026-B42] Revez J, Schott T, Llarena A-K, Rossi M, Hänninen M-L. 2013 Genetic heterogeneity of *Campylobacter jejuni* NCTC 11168 upon human infection. Infect Genet Evol. 16:305–309. 23523819 10.1016/j.meegid.2013.03.009

[evy026-B43] Richardson PT, Park SF. 1995 Enterochelin acquisition in *Campylobacter coli*: characterization of components of a binding-protein-dependent transport system. Microbiology 141 12:3181–3191.http://dx.doi.org/10.1099/13500872-141-12-3181 8574410 10.1099/13500872-141-12-3181

[evy026-B44] Rocha EP. 2004 Codon usage bias from tRNA's point of view: redundancy, specialization, and efficient decoding for translation optimization. Genome Res. 14 11:2279–2286.http://dx.doi.org/10.1101/gr.2896904 15479947 10.1101/gr.2896904 PMC525687

[evy026-B45] Sahin O, 2012 Molecular evidence for zoonotic transmission of an emergent, highly pathogenic *Campylobacter jejuni* clone in the United States. J Clin Microbiol. 50 3:680–687.http://dx.doi.org/10.1128/JCM.06167-11 22189122 10.1128/JCM.06167-11 PMC3295108

[evy026-B46] Sanad YM, 2011 Genotypic and phenotypic properties of cattle-associated *Campylobacter* and their implications to public health in the USA. PLoS One 6 10:e25778. 22046247 10.1371/journal.pone.0025778 PMC3198382

[evy026-B47] Seal BS, 2007 Proteomic analyses of a robust versus a poor chicken gastrointestinal colonizing isolate of *Campylobacter jejuni*. J Proteome Res. 6 12:4582–4591.http://dx.doi.org/10.1021/pr070356a 17973442 10.1021/pr070356a

[evy026-B48] Sheppard SK, Dallas JF, MacRae M, 2009 *Campylobacter* genotypes from food animals, environmental sources and clinical disease in Scotland 2005/6. Int J Food Microbiol. 134(1–2):96–103. 19269051 10.1016/j.ijfoodmicro.2009.02.010 PMC3985063

[evy026-B49] Sheppard SK, Dallas JF, Strachan NJ, 2009 *Campylobacter* genotyping to determine the source of human infection. Clin Infect Dis. 48 8:1072–1078.http://dx.doi.org/10.1086/597402 19275496 10.1086/597402 PMC3988352

[evy026-B50] Sheppard SK, Didelot X, Jolley KA, 2013 Progressive genome-wide introgression in agricultural *Campylobacter coli*. Mol Ecol. 22 4:1051–1064.http://dx.doi.org/10.1111/mec.12162 23279096 10.1111/mec.12162 PMC3749442

[evy026-B51] Sheppard SK, Didelot X, Meric G, 2013 Genome-wide association study identifies vitamin B5 biosynthesis as a host specificity factor in *Campylobacter*. Proc Natl Acad Sci U S A. 110 29:11923–11927. 23818615 10.1073/pnas.1305559110 PMC3718156

[evy026-B52] Sheppard SK, 2010 Host association of *Campylobacter* genotypes transcends geographic variation. Appl Environ Microbiol. 76 15:5269–5277.http://dx.doi.org/10.1128/AEM.00124-10 20525862 10.1128/AEM.00124-10 PMC2916502

[evy026-B53] Sheppard SK, 2011 Niche segregation and genetic structure of *Campylobacter jejuni* populations from wild and agricultural host species. Mol Ecol. 20 16:3484–3490.http://dx.doi.org/10.1111/j.1365-294X.2011.05179.x 21762392 10.1111/j.1365-294X.2011.05179.x PMC3985062

[evy026-B54] Sheppard SK, 2014 Cryptic ecology among host generalist *Campylobacter jejuni* in domestic animals. Mol Ecol. 23 10:2442–2451.http://dx.doi.org/10.1111/mec.12742 24689900 10.1111/mec.12742 PMC4237157

[evy026-B55] Sheppard SK, Jolley KA, Maiden MCJ. 2012 A gene-by-gene approach to bacterial population genomics: whole genome MLST of *Campylobacter*. Genes 3 4:261–277.http://dx.doi.org/10.3390/genes3020261 24704917 10.3390/genes3020261 PMC3902793

[evy026-B56] Skarp CPA, Hanninen ML, Rautelin HIK. 2016 Campylobacteriosis: the role of poultry meat. Clin Microbiol Infect. 22 2:103–109.http://dx.doi.org/10.1016/j.cmi.2015.11.019 26686808 10.1016/j.cmi.2015.11.019

[evy026-B57] Sopwith W, 2008 Identification of potential environmentally adapted *Campylobacter jejuni* strain, United Kingdom. Emerg Infect Dis. 14 11:1769–1773.http://dx.doi.org/10.3201/eid1411.071678 18976567 10.3201/eid1411.071678 PMC2630731

[evy026-B58] Stamatakis A. 2014 RAxML version 8: a tool for phylogenetic analysis and post-analysis of large phylogenies. Bioinformatics 30 9:1312–1313.http://dx.doi.org/10.1093/bioinformatics/btu033 24451623 10.1093/bioinformatics/btu033 PMC3998144

[evy026-B59] Tajima F, Nei M. 1984 Estimation of evolutionary distance between nucleotide sequences. Mol Biol Evol. 1 3:269–285. 6599968 10.1093/oxfordjournals.molbev.a040317

[evy026-B60] Tamura K, Kumar S. 2002 Evolutionary distance estimation under heterogeneous substitution pattern among lineages. Mol Biol Evol. 19 10:1727–1736.http://dx.doi.org/10.1093/oxfordjournals.molbev.a003995 12270899 10.1093/oxfordjournals.molbev.a003995

[evy026-B61] Tareen AM, Dasti JI, Zautner AE, Gross U, Lugert R. 2011 Sulphite: cytochrome c oxidoreductase deficiency in *Campylobacter jejuni* reduces motility, host cell adherence and invasion. Microbiology 157(Pt 6):1776–1785. 21372092 10.1099/mic.0.045567-0

[evy026-B62] Thepault A, 2017 Genome-wide identification of host-segregating epidemiological markers for source attribution in *Campylobacter jejuni*. Appl Environ Microbiol. 17;83 7:e03085–16. 28115376 10.1128/AEM.03085-16 PMC5359498

[evy026-B63] Thomas DK, 2014 Comparative variation within the genome of *Campylobacter jejuni* NCTC 11168 in human and murine hosts. PLoS One 9 2:e88229. 24516617 10.1371/journal.pone.0088229 PMC3917866

[evy026-B64] Thompson SA, Gaynor EC. 2008 *Campylobacter jejuni* host tissue tropism: a consequence of its low-carb lifestyle? Cell Host Microbe 4 5:409–410. 18996338 10.1016/j.chom.2008.10.010 PMC2762321

[evy026-B65] van der Stel AX, 2015 The *Campylobacter jejuni* RacRS system regulates fumarate utilization in a low oxygen environment. Environ Microbiol. 17 4:1049–1064.http://dx.doi.org/10.1111/1462-2920.12476 24707969 10.1111/1462-2920.12476

[evy026-B66] Vidal AB, 2016 Genetic diversity of *Campylobacter jejuni* and *Campylobacter coli* isolates from conventional broiler flocks and the impacts of sampling strategy and laboratory method. Appl Environ Microbiol. 82 8:2347–2355.http://dx.doi.org/10.1128/AEM.03693-15 26873321 10.1128/AEM.03693-15 PMC4959481

[evy026-B67] Weinert LA, 2015 Genomic signatures of human and animal disease in the zoonotic pathogen *Streptococcus suis*. Nat Commun. 6 1:6740. http://dx.doi.org/10.1038/ncomms7740 25824154 10.1038/ncomms7740 PMC4389249

[evy026-B68] Wimalarathna HM, 2013 Widespread acquisition of antimicrobial resistance among *Campylobacter* isolates from UK retail poultry and evidence for clonal expansion of resistant lineages. BMC Microbiol. 13:160. 23855904 10.1186/1471-2180-13-160 PMC3717071

[evy026-B69] Woodcock DJ, 2017 Genomic plasticity and rapid host switching can promote the evolution of generalism: a case study in the zoonotic pathogen Campylobacter. Sci Rep. 29;7(1):9650. 10.1038/s41598-017-09483-9 PMC5575054 28851932

[evy026-B70] Yahara K, 2017 Genome-wide association of functional traits linked with *Campylobacter jejuni* survival from farm to fork. Environ Microbiol. 19 1:361–380.http://dx.doi.org/10.1111/1462-2920.13628 27883255 10.1111/1462-2920.13628

[evy026-B71] Zerbino DR, Birney E. 2008 Velvet: algorithms for de novo short read assembly using de Bruijn graphs. Genome Res. 18 5:821–829.http://dx.doi.org/10.1101/gr.074492.107 18349386 10.1101/gr.074492.107 PMC2336801

