# Supplementary figures and images for: Convergent Amino Acid Signatures in Polyphyletic Campylobacter jejuni Subpopulations Suggest Human Niche Tropism
Source: Genome Biol Evol. 2018 Feb 14;10(3):763–74. doi: 10.1093/gbe/evy026 (PMC5841378; doi:10.1093/gbe/evy026)

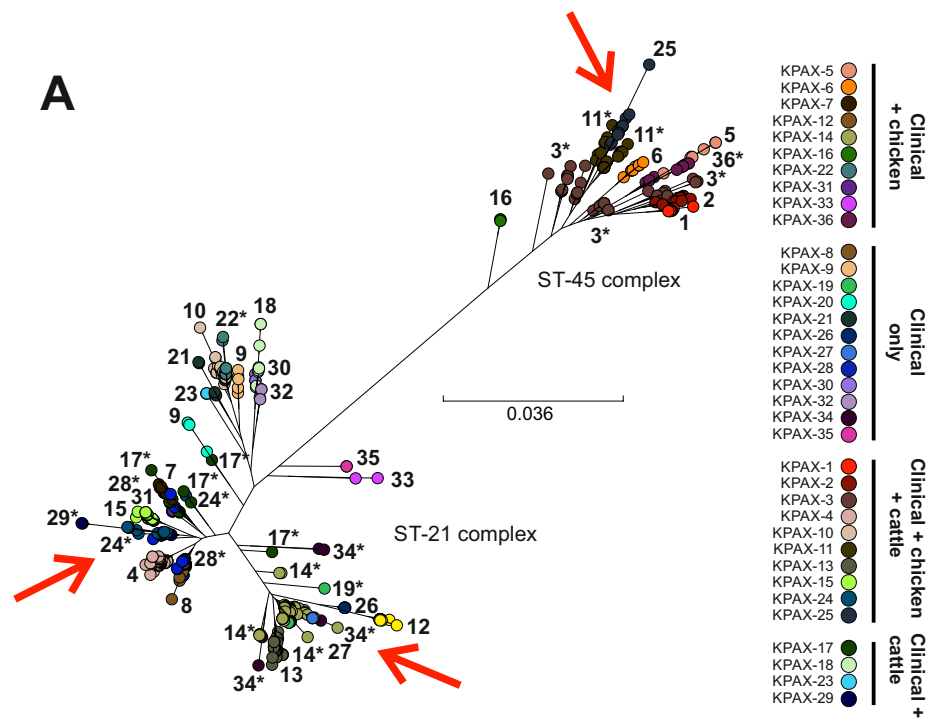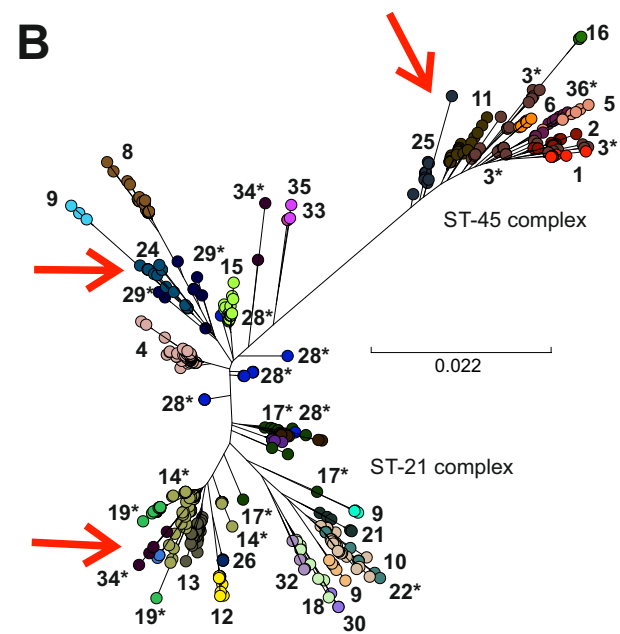

Supplement: Supplementary Data [file evy026_supp.zip › Figure_S1.pdf]
